# Design and Experimental Validation of a Novel Parallel Compliant Ankle for Quadruped Robots

**DOI:** 10.3390/biomimetics10100659

**Published:** 2025-10-01

**Authors:** Zisen Hua, Yongxiang Cheng, Xuewen Rong

**Affiliations:** 1 The First Affiliated Hospital, Anhui University of Science & Technology, Huainan 232001, China; 2 School of Artificial Intelligence, Anhui University of Science & Technology, Hefei 231131, China; 3 School of Control Science and Engineering, Shandong University, Jinan 250062, China

**Keywords:** quadruped robots, ankle structure, passive compliant

## Abstract

In this study, a novel compliant ankle structure with three passive degrees of freedom for quadruped robots is presented. First, this paper introduced the bionic principle and structural implementation method of the passively compliant ankle, with a particular focus on the configuration and working principle of the elastic adjustment element. Then, the kinematic model of the ankle and mathematic model of the elastic element, comprising mechanical and pneumatic model, was established by using appropriate theory. Finally, a test rig of the ankle was carried out to verify its actual function. The research results show that: (1) The ankle structure demonstrates excellent stability, maintaining its upright posture even under unreliable foot–ground interactions. (2) Compared to traditional structure, the single-leg module incorporating the proposed design exhibits smoother forward stepping under an appropriate pre-inflation pressure, with its actual motion trajectory showing closer agreement with the planned one; (3) The parallel topology enables a notable reduction in the driving torque of each joint in the leg during motion, thereby improving the energy efficiency of robots.

## 1. Introduction

Legged robots utilize discrete footholds during locomotion, offering superior adaptability to complex and uneven terrains. Their multi-limb, multi-degree-of-freedom (DOF) architecture not only facilitates flexible posture adjustments for task-specific demands but also provides inherent fault tolerance—enabling continued operation even in the event of failure in one or more limbs. Consequently, the development of legged robots with robust motion flexibility and exceptional terrain adaptability for operation is of great practical significance and holds broad potential for deployment in wild unstructured environments.

Currently, the predominant approach to achieving mammalian biomimicry in legged robots remains largely confined to simplified structural imitation. In particular, limb biomimicry research often limits itself to replicating the two- or three-segment leg bone configuration above the ankle joint observed in animals, rarely incorporating sophisticated mechanical architectures to emulate the full structural and functional complexity of biological limbs. Representative examples of such highly abstracted designs include MIT’s Cheetah3 [1], Boston Dynamics’ SpotMini, the Italian Institute of Technology’s HyQReal [2], Zhejiang University’s “Jueying” [3], and Unitree Robotics’ Go1 [4]. While this simplification helps reduce design and manufacturing complexity, it also inherently limits locomotion performance and adaptability to complex environments.

To enhance foot-ground compliance and achieve dynamically stable locomotion, researchers have made substantial progress in refining force control strategies and developing novel actuation methods, seeking to compensate for limitations in limb structural design through advanced control techniques. For instance, Liufeng Wang et al. [5] proposed a dynamic balance control framework based on QP (Quadratic Programming) and impedance control. Guangrong Chen et al. [6] presented a Virtual Model Control (VMC) method to achieve dynamic balance control for quadruped robots during trot gait. Jinwoo Jeon et al. [7] introduced FLONIC-Q: an intelligent control system based on Fuzzy Logic and Neural Networks for quadruped robots operating on extreme terrain. To improve the stability and environmental adaptability of quadruped robots during trotting gait, Baoping Ma et al. [8] proposed a behavior control method based on swing leg gait planning. Zhang et al. [9] presented an end-to-end reinforcement learning (RL) strategy, integrating proprioception and exteroception, to handle perception failures. When quantitatively compared against existing heuristic-based local reactive planners, their strategy achieved over 30% higher success rates when facing perception faults. Chen et al. [10] proposed an RGB-D system integrated with the Model Predictive Control (MPC) and Whole-Body Control (WBC) of the MIT Cheetah for dynamic path planning. The results demonstrated that, compared to a SLAM system, the RGB-D system achieved a 20% improvement in velocity stability and a 10% improvement in trajectory accuracy; in complex environments, it also exhibited a 15% reduction in path planning iterations and a 30% increase in recovery speed. Takahiro Miki et al. [11] employed an attention mechanism to fuse exteroception and proprioception, enhancing the operational speed and stability of a quadruped robot in field environments. Zhang et al. [12] combined MPC with RL, utilizing PD-based MPC to generate samples and then optimizing parameters online using reinforcement learning. Compared to an MPC controller with fixed parameters, this approach achieved significant improvements in both locomotion performance and stability. Although this methodology of enhancing robotic mobility via algorithmic optimization has improved locomotion performance to some extent, it still falls short of entirely meeting the locomotion demands of complex environments.

In nature, the adaptive limb behavior of quadruped mammals in response to environmental cues does not arise solely from centralized control, particularly in the foot-ankle region. During limb posture transitions, the ankle often exhibits a high degree of passive compliance, which plays a critical role in maintaining postural stability during dynamic motion, dissipating foot-ground impact forces, distributing support loads, and perceiving the “shape-mechanical” properties of the supporting surface. Therefore, enhancing structural and functional biomimicry in robotic limbs through synergistic coordination of multi-segment limbs and compliant joint configurations is essential to address current limitations in robotic mobility and stability. Alexander Spröwitz et al. [13] designed the quadruped robot Cheetah-cub, characterized by its spring-embedded compliant legs, achieving the highest Froude number (1.42 m/s) for its time during trotting gait. Cheetah-Cub-S [14] introduced a compliant spine to the original Cheetah-cub design, reducing its minimum turning radius to 0.51 m. Kazuhiro Miyashita et al. [15] biomimetically replicated the muscle-tendon structure of horse limbs, enabling a leg mechanism that could autonomously generate stable walking and smooth swing-stance transitions through simple sinusoidal hip actuation. However, the aforementioned three designs are confined to a single degree of freedom, rendering them inadequate for adapting to complex and varied terrains. Moreover, the utilization of rudimentary elastic components, such as coil springs or elastic cords, significantly constrains the load-bearing capacity of the robot. Fangyan Shen et al. [16] designed a foot-ankle module for bipedal robots, demonstrating effective impact energy dissipation, slope adaptability, and robustness to ground unevenness. Huang et al. [17] proposed a novel 3D passively compliant leg structure that was low-cost, lightweight, and featured tunable stiffness. Both simulation and hardware tests confirmed its stability and impact resistance. Jiang et al. [18] developed a pneumatically actuated soft–rigid hybrid joint for quadruped robots, combining the advantages of elastic compliance and rigid articulation to enhance joint responsiveness and collision buffering. F. Bjelonic et al. [19] performed co-design optimization of parallel elastic joints for the ANYmal quadruped robot, increasing the robot’s torque-squared efficiency by 33%, reducing joint torque by 30%, and extending operational time. Jiang et al. [20] compared the dynamics of rigid versus compliant spines during robot aerial motions; their study indicated that the compliant spine significantly enhanced jump height, horizontal velocity, and landing stability. Singh S et al. [21] introduced a “C-groove” compliant leg segment design, which, in simulations, led to more than 70% reduction in peak joint torques across all cases. Sun et al. [22] proposed a multi-objective topology optimization approach for compliant leg structures in bionic quadrupeds. Experimental validation showed that the robot could perform a biologically inspired two-phase bending gait, demonstrating excellent straight-line walking capability and carrying loads up to eight times its own weight. However, although the adoption of elastomers with specifically shaped small-deformations to enhance the flexibility of the ankle joint, or the utilization of upper joint flexibility to indirectly achieve passive adaptability of the foot to external environments, has demonstrated significant effectiveness in foot-ground impact absorption and energy efficiency, these approaches have not fundamentally and comprehensively improved the contact state between the foot and the ground. Consequently, they fail to generate systematic dynamic effects on the robot. The rational evolution from rigid joints to flexible joints is an inevitable trend, but the core of biomechanical motion lies in the scientific matching of degrees of freedom and flexibility.

In summary, although the ankle joint plays a crucial role in generating efficient and flexible adaptive movements in complex environments, it has not been effectively emphasized in the current structural biomimicry of quadrupedal robots. The purpose of this paper is to achieve a deep bionic imitation of the ankle structure of canine mammals, thereby enabling a quadruped robot to preliminarily acquire the capability to adaptively adjust its foot-ground posture in response to the morphology of the support domain. This aims to enhance locomotion stability and enable terrain perception. To this end, this paper presents a novel compliant ankle structure for quadruped robots. By introducing a 3-CUU-S (C: cylinder joint; U: universal joint; S: spherical joint) topological configuration and leveraging a unique elastic element design, the design enables passive three-degree-of-freedom adjustment of the footplate relative to the ground. Meanwhile, the pneumatic-driven elastic telescopic design results in a more compact structure that better satisfies the dynamic actuation demands of quadruped robots, and allows the adjustment force for footplate posture to be optimized according to the load. Hence, this structure not only contributes to a significant improvement in the locomotion stability of the robot but also exerts a positive effect on the enhancement of the motion energy efficiency.

The specific structure of the paper is as follows: Section 2 mainly describes the bionic principle and the detailed structure of the passively compliant ankle; Section 3 derives the kinematic model of the structure and analyzes the dynamic output characteristics of the elastic element. Section 4 gives the experiment verification results. At last, Section 5 presents the conclusions and outlines future research directions.

## 2. Structural Design of Passively Compliant Ankle

### 2.1. Analysis of Bionic Design Principles

**Figure 1:**
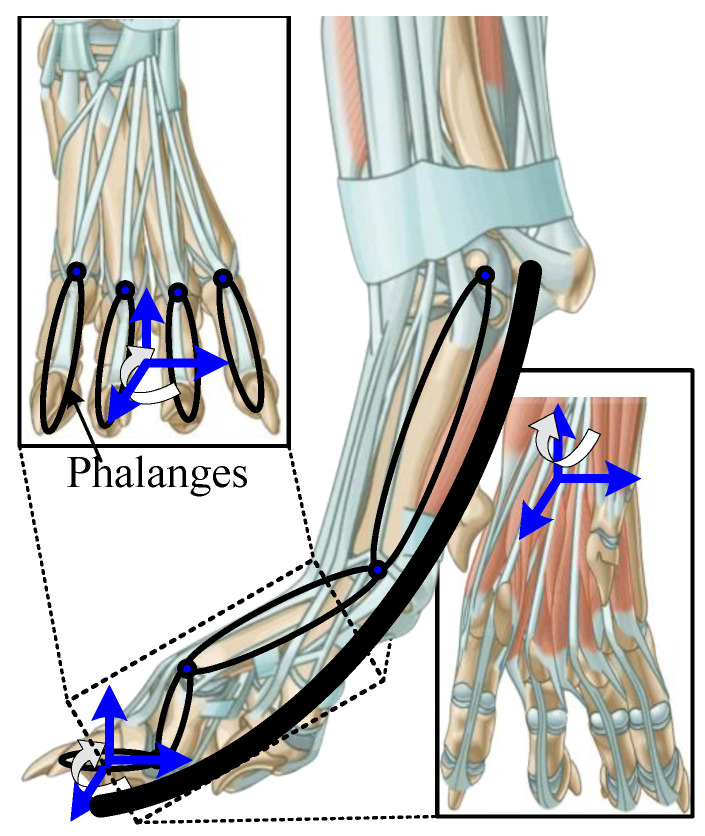
Physiological structure of the ankle joint in canine mammals.

Quadruped mammals in nature can be broadly classified into three locomotion postures: plantigrade, digitigrade, and unguligrade. Compared to the plantigrade gait exemplified by primates, the digitigrade gait, typical of canids, makes ground contact using only the distal phalanges, shown in the Figure 1. This configuration shortens the transition time from initial contact to full foot placement and reduces the need for foot posture adjustment. Concurrently, the inherently unstable toe-contact structure causes the foot to behave like an inverted arch functioning as a compression spring. This morphology provides effective impact absorption and energy storage, supporting high-powered jumping and rapid running. Additionally, the separated, multi-jointed digital configuration also enables tactile perception of the external morphology within the local support zone beneath the foot. Such structurally coordinated compliance, inherently embedded in biological feet, continues to pose a significant challenge for replication in current robotic or mechanical analogs.

Unlike bipeded robots, quadruped robots primarily maintain dynamic balance through distributed multi-point contact. The prevailing design paradigm, featuring rigid limb segments combined with a compliant foot sole, eliminates the need for secondary foot posture adjustments after ground contact, thereby facilitating faster recovery from instability. However, the absence of passive compliance to accommodate micro-terrain variations deprives the robot of the ability to directly perceive the external geometry and intrinsic physical properties of the support surface. Moreover, such designs offer no direct or effective response to several critical challenges: high-impact forces caused by model inaccuracies, low control bandwidth, or component wear; and fine-grained posture adjustments required to resist micro-slippage or prevent contact failure under marginally stable conditions. These adaptive capabilities cannot be achieved through active control alone.

### 2.2. Theoretical Analysis and Structural Design

**Figure 2:**
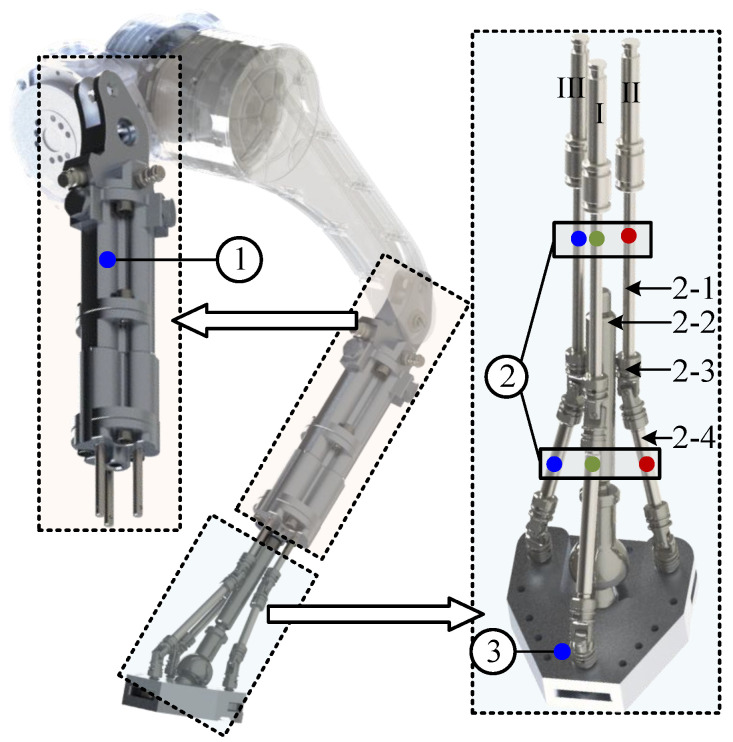
Passively compliant bionic foot-ankle with 3-CUU-S type.

Based on the preceding analysis, to replicate the foot-ground interaction mode of digitigrade legged mammals and enable deep perception of terrain morphology at the support interface, the key challenge lies in transforming the conventional rigid foot–ankle structure into a flexible system with inherent passive compliance. This transformation hinges on a rational structural layout and appropriate degree-of-freedom (DOF) configuration. To this end, drawing upon the design methodology of a master 6-DOF parallel platform, this paper proposes a 3-CUU-S topology for a passively compliant biomimetic foot–ankle mechanism, achieved through optimized linkage arrangement and DOF configuration, as illustrated in Figure 2.

The proposed design consists of three major components: an upper linear-motion elastic element, a middle transmission linkage assembly, and a bottom foot segment. The intermediate linkage chain employs Universal (U) joints (2-3) to transmit bidirectional adjustment forces and posture variations between the foot segment and the elastic actuator. The primary supporting rod (2-2), rigidly connected to the actuator, is linked to the foot via a Spherical (S) joint and is responsible for bearing the robot’s vertical load after foot-ground contact is established. Considering the symmetry of lateral sway motion, Linkage Chains II and III, connected to the posterior sides of the footplate via spherical joints, are arranged in a left–right symmetric configuration. In contrast, Linkage Chain I connects to the anterior part of the footplate through a front-centered spherical joint. According to the Kutzbach–Grübler criterion for spatial mechanisms, this configuration endows the foot segment with three rotational DOFs, which are sufficient to meet the requirements for compliant foot posture adjustment during foot-ground interaction.

**Figure 3:**
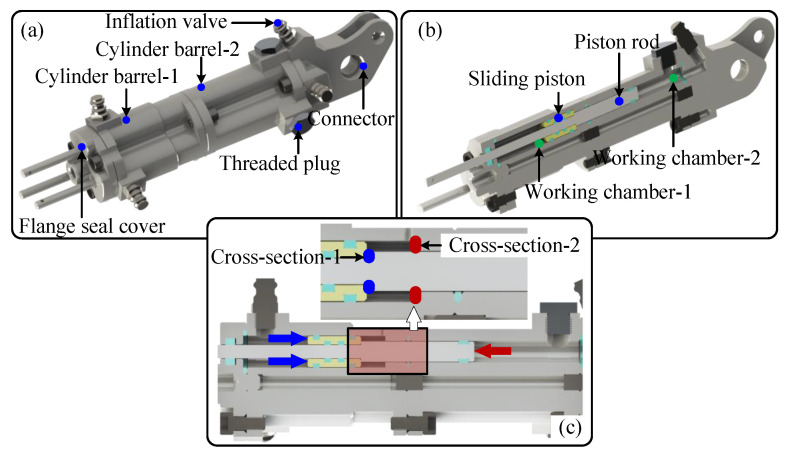
Compact triple-piston passively elastic element. (**a**) 3D structure; (**b**) Cross-sectional view; (**c**) Refined structure of the working chamber.

To effectively expand the range of foot posture adjustment while adhering to integration and lightweight design principles, the linear-motion elastic element adopts a pneumatic actuation scheme, as illustrated in Figure 3a. The pneumatic support cylinder features a modular design with three piston chambers uniformly arranged in a circumferential layout. Notably, the piston chamber within cylinder barrel-1 has a slightly larger diameter than that in cylinder barrel-2 (see Figure 3b). The small diameter section of the piston rod is fitted with a sliding piston which moves solely within the piston chamber of cylinder barrel-1. Conversely, its large diameter section extends directly into the working chamber of cylinder barrel-2. Consequently, taking the illustrated cross-section as an example, this pneumatic support system comprises two distinct working chambers:

- Working chamber-1: Defined between the end face of the sealing cap and the sliding piston.
- Working chamber-2: Defined between the end face of the piston shaft and the end face of the connector.

Miniature one-way inflation valves are integrated at the respective end positions of each chamber, allowing flexible regulation of the pre-charge pressure within each chamber to accommodate varying operational requirements. As illustrated in Figure 3c, the fundamental operating principles of this structure can be summarized as follows:

(a) Neutral Position (No External Load): When no external load is applied to the piston rod, the pre-charge pressures in both working chambers can be appropriately adjusted such that cross-section-1 and cross-section-2 are aligned. In this state, the compressive force generated by the sliding piston suspends the piston rod at a neutral position.
(b) Tension Mode (Tensile Force Applied): When a tensile force is applied to the piston rod, cross-section-1 comes into contact with the sliding piston, pushing it backward. As a result:
  - The volume of Working Chamber-1 decreases, causing its internal pressure to rise.
  - The volume of Working Chamber-2 increases, leading to a drop in pressure.
  - The resulting net force still acts to restore the piston rod toward its neutral position.
(c) Compression Mode (Compressive Force Applied): When a compressive force is applied to the piston rod, cross-section-2 constrains the sliding piston, preventing it from moving. In this condition, the piston rod retracts independently, compressing the gas within Working Chamber 2. Meanwhile, the volume of Working Chamber-1 remains unchanged, and its internal pressure is thus maintained at a steady level.

## 3. Modeling and Analysis

This section investigates in-depth the mapping between linkage branch deformations and footplate pose based on the proposed structural kinematic model, while also analyzing the rationale for determining the initial inclination angle of the footplate relative to the primary support. Furthermore, the dynamic actuation characteristics of the elastic element are clarified through pneumatic principles.

### 3.1. Relationship Between Footplate Pose and Elastic Element Deformation

**Figure 4:**
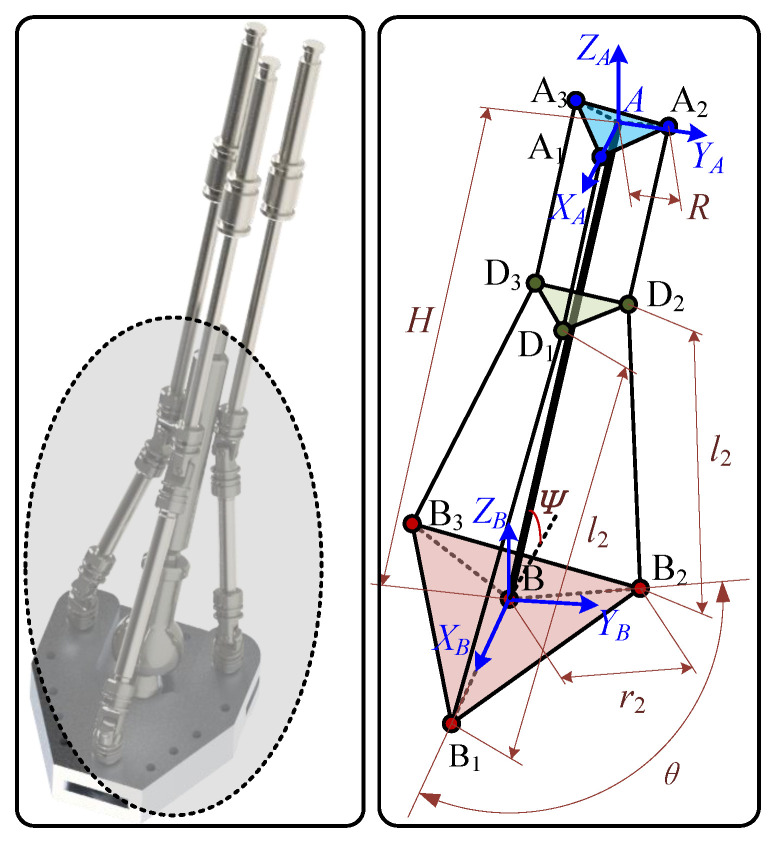
Schematic diagram of the foot–ankle structure with node definitions.

Based on the topological design of the compliant foot–ankle structure, the system can be simplified into a spatial model composed of one variable plane D_1_D_2_D_3_ and fixed planes A_1_A_2_A_3_ and B_1_B_2_B_3_, as illustrated in Figure 4. The coordinate system {A-X_*A*_Y_*A*_Z_*A*_} is defined with its origin at the centroid of the triangle A_1_A_2_A_3_, whose vertices are evenly distributed along a circular arc with radius *R*. The Z_*A*_-axis is normal to the X_*A*_Y_*A*_ plane. The coordinate system {B-X_*B*_Y_*B*_Z_*B*_} is defined with its origin located at the spherical joint on the footplate, and its Z_*B*_-axis is also normal to the footplate. Ψ denotes the inherent structural angle between the footplate and the primary support rod AB. In the initial configuration, the X_*B*_ and Y_*B*_-axes of the footplate coordinate system are parallel to those of the base coordinate system. The lengths of segments BB_*i*_ and B_*i*_D_*i*_ (for i=1,2,3) are denoted as $r_{i}$ and $l_{i}$, respectively. The length of segment AB is denoted as *H*. The angles between BB_*i*_ and BB_1_, as well as between AA_*i*_ and AA_1_, are denoted as $\theta_{i}^{B}$ and $\theta_{i}^{A}$, respectively.

The rotation matrix of coordinate system {B-X_*B*_Y_*B*_Z_*B*_} relative to {A-X_*A*_Y_*A*_Z_*A*_} can be expressed as:

(1) RotBA=rotz(γ)roty(β)rotx(α)

The position of point B_*i*_ in the coordinate system {B-X_*B*_Y_*B*_Z_*B*_} is given by the vector [$x_{i}^{B}$, $y_{i}^{B}$,0], and can be expressed as:

(2) xiB=ricosθiByiB=risinθiB

Therefore, combining Formulas (1) and (2), the position of point B_*i*_ in coordinate system {A-X_*A*_Y_*A*_Z_*A*_} can be expressed as:

(3) BiA=RotBA·BiB+BTA

where

(4) BA=HcosΨ0−HsinΨ

Given that hinge point D and point A lie on the same axis, the position of point D in the coordinate system {B-X_*B*_Y_*B*_Z_*B*_} is given by:

(5) xiDA=RcosθiAyiDA=RsinθiAziDA=di+Δi

where $d_{i}$ represents the displacement of piston *i* along the Z_*A*_ axis direction, and $\Delta_{i}$ is the initial distance between A_*i*_ and D_*i*_.

Considering that the length of B_*i*_D_*i*_ is a definite quantity, it satisfies:

(6) xiA−xiD2+yiA−yiD2+ziA−ziD2=Li2

To this end, the mapping relationship between the displacement $d_{i}$ of piston *i* and the angle of the footplate can be obtained as follows:

(7) di=ziBA+Li2−xiBA−xiDA2−yiBA−yiDA2−Δi

The main parameters involved in the above formulas are shown in Table 1, and their specific values can be obtained through measurements performed in 3D modeling software.

**Figure 5:**
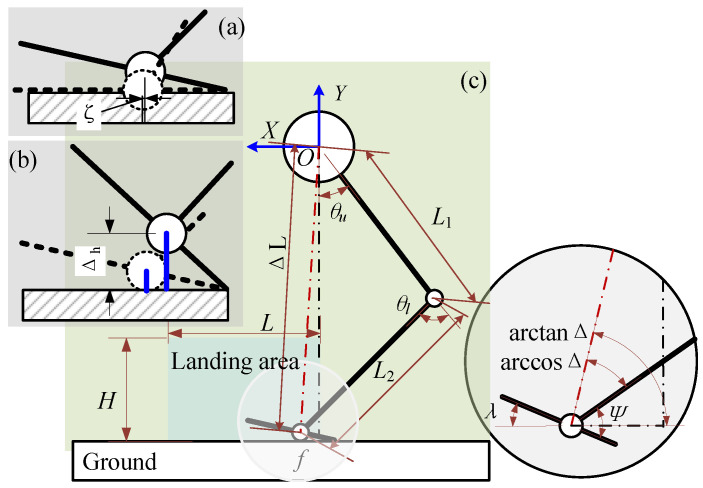
Schematic of the relationship and value principle between angle λ and Angle Ψ. (**a**) Horizontal position change of footplate spherical joint center during foot-ground contact; (**b**) Vertical position change of footplate spherical joint center during foot-ground contact. (**c**) Structural and kinematic parameters definition for the single leg.

Based on the geometric relationships described above, the deformation behavior of the elastic element is influenced not only by variations in footplate pose, but also by the initial inclination angle Ψ between the footplate and the primary supporting rod. As illustrated in Figure 5, for forward locomotion to proceed without disruption to the horizontal velocity of the shoulder joint reference point *O*, the footplate-ground inclination angle (λ) must remain positive (λ>0) throughout the transition from initial contact to full foot support within the available foothold region. However, if the inclination angle λ becomes excessively large, it will induce greater vertical fluctuations in the robot’s center of mass due to the increased vertical displacement component during foot placement. To quantitatively describe this relationship, the dynamic formulation of the footplate-ground inclination angle λ is derived based on the parameter definitions provided in Figure 5, as follows:

(8) $$\lambda =\Psi +\arctan\frac{y_{f}}{x_{f}}+\arccos\frac{L_{2}^{2}+\Delta L^{2}-L_{1}^{2}}{2\Delta LL_{2}}$$

where $x_{f}$ and $y_{f}$ represent the position of the foot endpoint in the reference coordinate system {O-XY}, $L_{1}$ and $L_{2}$ represent the length of the thigh link and calf link, respectively; ΔL is the distance from the foot endpoint to the origin of the coordinate system, and can be expressed as:

(9) $$\Delta L=\sqrt{x_{f}^{2}+y_{f}^{2}}$$

For a two-segment limb topology, greater standing height or step length will lead to a larger footplate-ground inclination angle λ. Using a nominal standing height of 400mm and setting λ=0 when the footplate is directly beneath the shoulder joint, the initial inclination angle Ψ calculated from Equation (9) is $57.32^{\circ }$.

**Figure 6:**
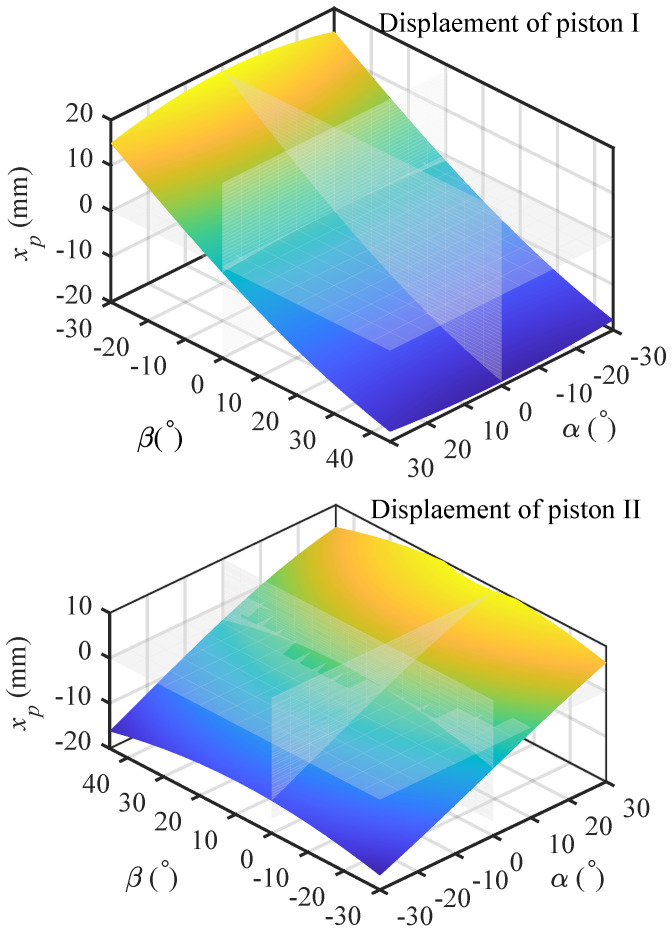
Correlation between piston stroke and footplate inclination.

Based on the aforementioned calculated values of Ψ, Figure 6 illustrates the relationship between the positional variation of the three piston axes and the pitch angle α and yaw angle β of the footplate within the conventional operational range of the spherical joint. Given the symmetric motion characteristics between Piston II and Piston III, only the data for Piston I and Piston II are presented. The results demonstrate that, under the current topological configuration, the linkage associated with Piston I predominantly governs the pitch adjustment of the footplate. Within the examined pitch range of −$30^{\circ }$ to $50^{\circ }$ (referenced to the coordinate system in Figure 4), the displacement of Piston I varies approximately linearly with the pitch angle α, ranging from −19.03 mm to 19.03 mm. In contrast, the linkage chains of Piston II and Piston III contribute to both pitch and roll adjustments. Within the evaluated range of −$30^{\circ }$ to $30^{\circ }$, their displacements are confined between −19 mm to 12 mm.

**Figure 7:**
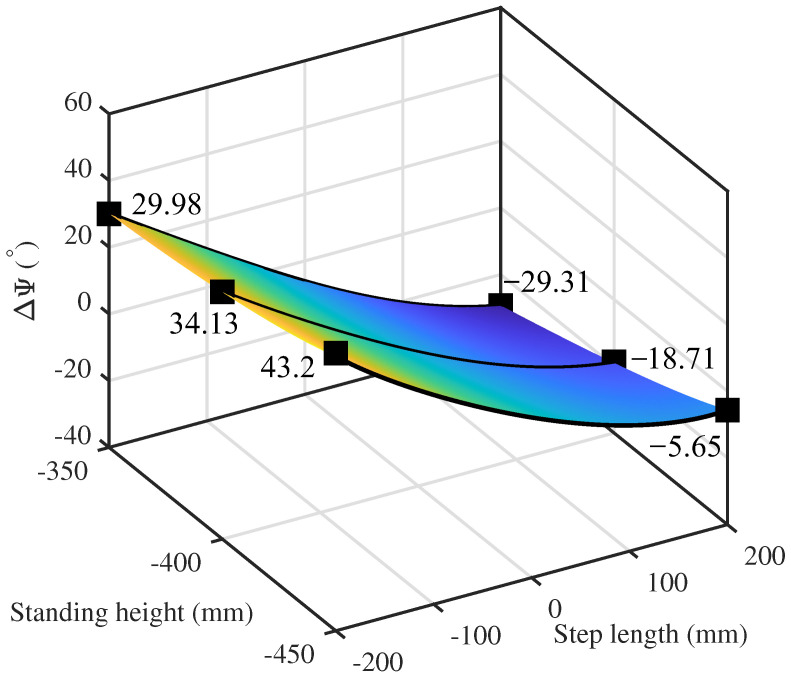
Dependence of the angle between primary support rod and footplate on step length and stance height.

Figure 7 depicts the influence of stance height and stride length on the angle Ψ throughout one supporting phase. To highlight the variation trends, curves for stance heights of 350 mm, 400 mm, and 450 mm are emphasized. As shown in the figure, throughout the entire motion from foot landing (200 mm) to foot lifting (−200 mm), the primary supporting rod continuously rotates counterclockwise around the spherical joint. With increasing stance height, both the compression magnitude of ΔΨ (Negative values correspond to a reduction in Ψ) and its variation range decrease. Nevertheless, the maximum pitch angle remains confined within $45^{\circ }$.

### 3.2. Dynamic Characteristics of the Elastic Element

**Figure 8:**
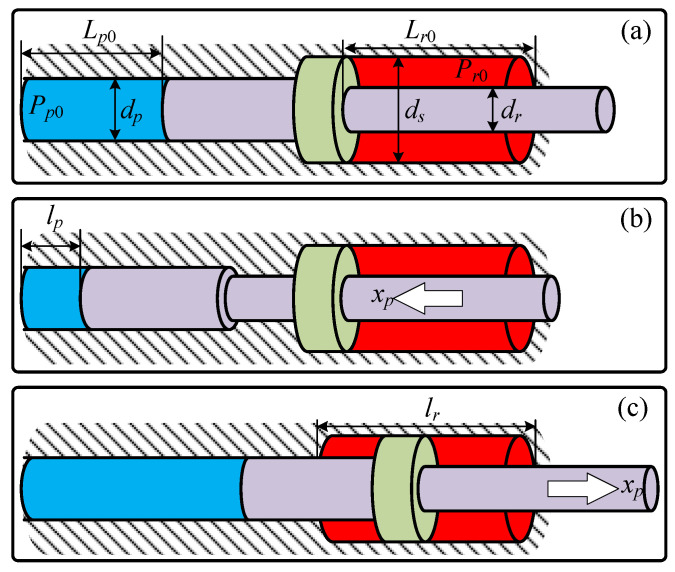
Operating principle of elastic element. (**a**) Structural parameters definition; (**b**) Piston retraction stroke; (**c**) Piston extension stroke.

Given that the three branches of the elastic element exhibit structurally identical configurations, any branch can be for analysis without loss of generality. As illustrated in Figure 8, the dynamic behavior during the piston’s extension and retraction can be characterized by the following mathematical formulations:

(10) $$\left\{\begin{array}{cc} m{\ddot{x}}_{p}=P_{p}A_{p}{-}^{e}f_{f}+f_{ext}\; & \left(x_{p}⩽L_{p0}\&{\dot{x}}_{p}>0\right)\\ m{\ddot{x}}_{p}=-P_{r}A_{r}+P_{p}A_{p}{-}^{r}f_{f}+f_{ext}\; & \left(L_{p0}<x_{p}⩽L_{p0}+L_{r0}\&{\dot{x}}_{p}>0\right) \end{array}\right.$$

(11) $$\left\{\begin{array}{cc} m{\ddot{x}}_{p}=P_{r}A_{r}-P_{p}A_{p}{-}^{r}f_{f}+f_{ext}\; & \left(L_{p0}<x_{p}⩽L_{p0}+L_{r0}\&{\dot{x}}_{p}<0\right)\\ m{\ddot{x}}_{p}=-P_{p}A_{p}{-}^{e}f_{f}+f_{ext}\; & \left(x_{p}⩽L_{p0}\&{\dot{x}}_{p}<0\right) \end{array}\right.$$

where *P* denotes the hydrodynamic pressure within the working chambers, with subscripts *r* and *p* specifically designating working chamber-1 and working chamber-2; $f_{ext}$ signifies the externally applied load force; $L_{p0}$ and $L_{r0}$ denote the total strokes of the pistons on both sides, respectively.

ffe and ffr represent the interfacial friction-damping behavior between piston assembly and cylinder barrel during extension and retraction strokes, governed by the Stribeck friction formulation:

(12) $$f_{f}=\left[F_{c}+\left(F_{s}-F_{c}\right)e^{-{\left(\frac{{\dot{x}}_{p}}{V_{s}}\right)}^{\delta }}\right]\cdot sign\left({\dot{x}}_{p}\right)+\sigma_{v}{\dot{x}}_{p}$$

where $F_{c}$ and $F_{s}$ denote the coulomb friction force and static friction force, respectively; $V_{s}$ is the stribeck velocity; ϕ is the decay exponent governing the transition regime; $\delta_{v}$ signifies the viscous friction coefficient; And ${\dot{x}}_{p}$ refers to the relative velocity between the piston rod and the chamber.

Based on Boyle’s Law for ideal gases, the dynamic evolution of chamber under isothermal conditions can be formulated as:

(13) $$\left\{\begin{array}{cc} {\dot{P}}_{p}=\frac{L_{p0}P_{p0}{\dot{x}}_{p}}{{\left(L_{p0}-x_{p}\right)}^{2}}\; & \left(x_{p}⩽L_{p0}+L_{r0}\right)\\ {\dot{P}}_{r}=0\; & \left(x_{p}⩽L_{p0}\right)\\ {\dot{P}}_{r}=\frac{L_{r0}P_{(}r0){\dot{x}}_{p}}{{\left(L_{p0}+L_{r0}-x_{p}\right)}^{2}}\; & \left(L_{p0}<x_{p}⩽L_{p0}+L_{r0}\right) \end{array}\right.$$

The critical structural parameters of the elastic element are summarized in Table 2, while the remaining parameters in the model adopt values from publicly available test data of conventional pneumatic actuators.

**Figure 9:**
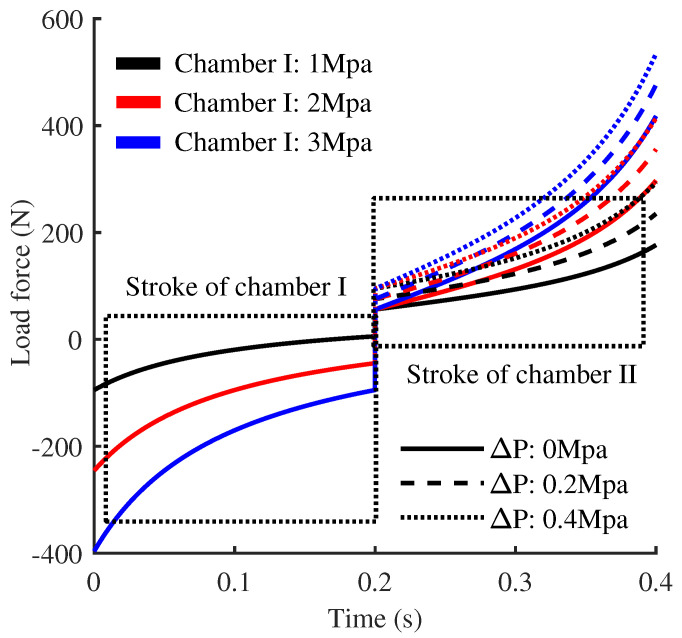
Relationship between output force and piston motion at varying initial inflation pressures.

Based on the aforementioned model, Figure 9 illustrates the variation curve of the theoretical traction force required to maintain piston extension at a constant velocity of 0.1 m/s. The pre-pressurization pressures for working chamber-1 are set at {1,2,3} MPa, while working chamber-2 follows the relation $P_{r0}=A_{p}/A_{r}\left(P_{p0}+\Delta \right)$. The actual test strokes of both chambers are constrained to 2/3 of full stroke to comply with safety regulations for high-pressure vessels in practical applications.

The results indicate that, when the piston transitions from retraction to extension from its stationary position, a notable jump in the elastic restoring force of the piston is observed. This phenomenon is attributed to the gas compression resistance within working chamber-2, where the sliding piston is housed. The magnitude of this jump exhibits a linear dependence on the inflation pressure, with higher pressures resulting in more pronounced effects. Furthermore, due to the larger effective force-bearing area of the sliding piston, the rate of change in the traction forces on either side of the stationary position is asymmetrical. Specifically, force variation rate accelerates significantly once the sliding piston is engaged in motion. In practical scenarios, this jump phenomenon is unavoidable, as it is influenced by uncertainties in the robot’s stance height and posture during operation.

## 4. Experimental Studies and Analysis

This section employs a single-leg experimental platform to further verify the influence of the novel structure on the robot’s conventional motion.

### 4.1. Overview of the Experimental Platform

**Figure 10:**
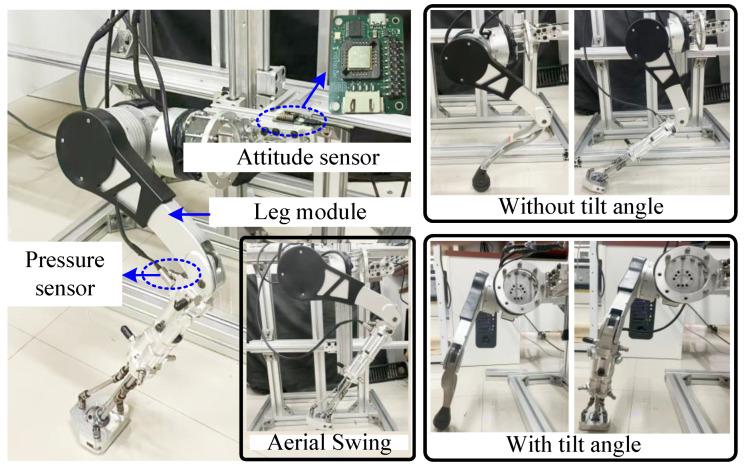
Single leg platform and experimental procedure.

Given that the lateral motion in practical applications serves only to maintain the robot’s stability and lacks periodicity, this study focuses exclusively on the periodic forward motion of the robot. To validate the feasibility of the new structure on practical applications, a single-leg experimental platform with two degrees of freedom was designed, as shown in Figure 10. The specific structural and dynamic parameters of the single-leg module in the comparison-group can be found in previous studies [23]. The effective length of the new structure and the dimensions of the joint interface are referenced to that of the comparison-group, with a total weight of approximately 1.1 kg. By employing linear bearings with low inertia, low damping, and low friction, the module is capable of free motion in both the vertical and horizontal directions within a plane. The total mass of the moving components in the platform is approximately 9 kg.

To precisely monitor the motion trajectories of reference points and evaluate the stability of the footplate during the experimental process, the miniature pressure sensors (Model: EB1UP-00000N-020BA) and the attitude sensors (Model: YESENCE106) are strategically installed at the upper end of the lateral swing motor connector and within the working chamber-2 of the front-end linkage, respectively. The pressure sensor signal is captured using an NI9220 16-bit data acquisition card operating at a sampling frequency of 1 kHz, and subsequently transmitted to the host computer for further analysis. And The pose sensor data can be directly read by the host computer.

### 4.2. Experimental Setup

The experiment primarily aims to assess the pose-locking capability of the elastomer within the new structure under no-load conditions, as well as to examine the impact of the new structure on the interaction stability and the dynamic motion stability of the robot during the support phase of periodic gait. Accordingly, two sub-experiments were conducted as follows:

- Step 1: With the single-leg module suspended and its planar degrees of freedom locked, high-frequency oscillations were applied to examine the effect of inflation pressure in the working chamber on the pose of the footplate.
- Step 2: With the single-leg module maintained at a constant standing height and in contact with the ground, horizontal stepping motions were performed to evaluate the influence of the structure on the velocity of the observation point in both the horizontal and vertical directions, while simultaneously monitoring the changes in the footplate–ground contact state.

### 4.3. Results and Discussions

**Figure 11:**
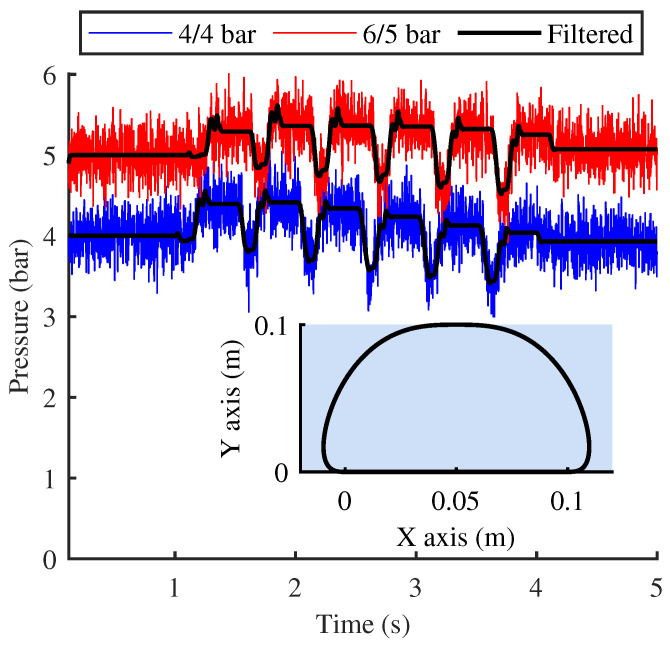
Oscillation of chamber pressure during the free swing phase.

Figure 11 illustrates the fluctuation data recorded by the pressure sensor during the first phase, where the single-leg module oscillates along a predefined trajectory at a frequency of 2 Hz. And the lateral swing motor remains in the zero position, ensuring that the entire single-leg module oscillates solely within the vertical plane in a forward and backward manner. The experiment is conducted in two groups, with the initial inflation pressures of the two working chambers (1 and 2) in the three linkage chains set to 4/4 bar and 5/6 bar, respectively. The selection of these pressure values was not based on specific criteria, as long as they were adequate to overcome internal resistance and ensure sufficient driving force. Through iterative adjustments and comparative experiments, it was found that when the inflation pressure is below 4 bar, the footplate exhibits significant oscillations during high-speed swinging. Conversely, when the inflation pressure exceeds 8 bar, the footplate fails to maintain stable contact with the ground in certain postures due to the relatively light mass of the single-leg model. Therefore, the inflation pressure range mentioned above was ultimately selected as the experimental condition.

The experimental results reveal that, under the predefined inflation pressure configurations, the pressure within the working chamber of the elastic element exhibits discernible fluctuations attributable to motion inertia. Specifically, the pressure oscillates within ranges of 3.44 to 4.4 bar and 4.6 to 5.5 bar for the two experimental groups, respectively. In accordance with Boyle’s gas law, these pressure variations correspond to piston displacement fluctuations of approximately −3 to 4 mm and −2 to 2 mm. By extrapolating these data through the motion model, the inclination angle of the footplate is determined to fluctuate within approximate ranges of $5^{\circ }$ and $4^{\circ }$ for the respective groups. Moreover, in the case of the 4/4 bar inflation configuration, the pressure fluctuations fail to demonstrate any discernible periodicity in response to the cyclic motion, with the pressure exhibiting a slight deviation from its baseline value once the oscillation ceased. From the filtered sensor data, it can be observed that, compared to the stable pressure value (4 bar) during the initial stationary phase, the stable pressure after motion stops has decreased by 5%. This phenomenon primarily arises from the friction and damping between the piston and the cylinder wall, with the reduced charging pressure being inadequate to effectively counteract these influences. Overall, the elastic element demonstrates a highly effective performance in stabilizing and locking the pose of the footplate when the inflation pressure is maintained at 6/5 bar, with a deviation in front and rear stabilization pressure of only 1.6%.

**Figure 12:**
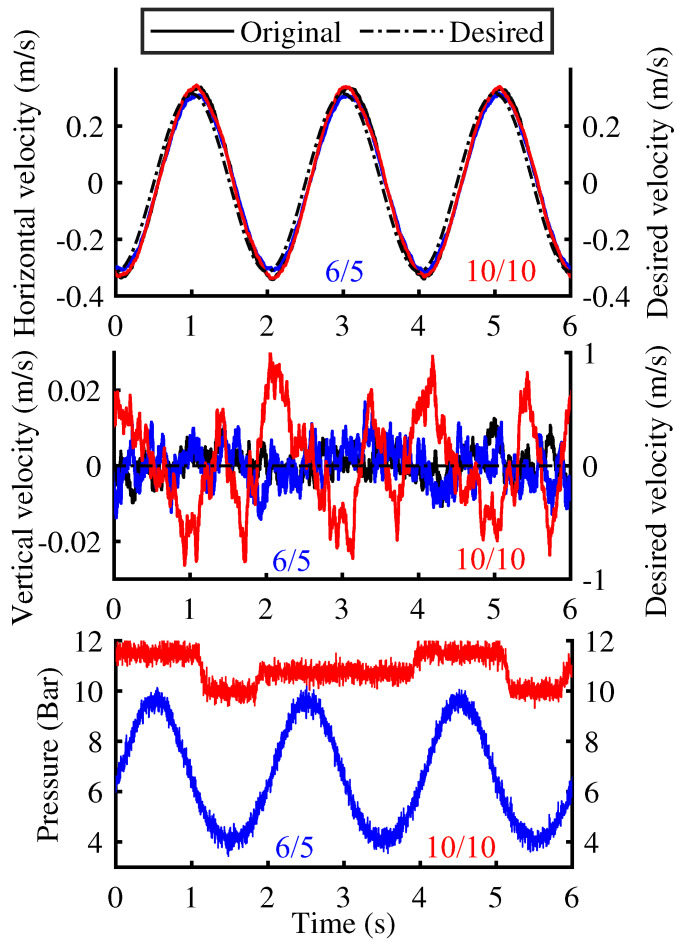
Dynamic response of observation point velocity and chamber pressure in the absence of limb lateral tilt.

Figure 12 illustrates the attitude and pressure sensor data collected during the second stage, in which the single-leg module executed horizontal stepping with an amplitude of 0.1 m at a frequency of 0.5 Hz. The curves in the figure are distinguished by different color-coded numerical combinations (for example, 6/5 indicates that the inflation pressures of working chambers 1 and 2 are 6 and 5 bar, respectively). Similarly, the entire single-leg module maintained motion within the sagittal plane. To enhance the comparative analysis, a test group employing a conventional lower-leg structure was also included. During the experiments, the inflation pressures in working chambers 1 and 2 were set to 6/5 and 10/10 bar, respectively.

A comprehensive comparison of the velocity and pressure data reveals that, at an inflation pressure of 6/5 bar, the observation point achieves superior velocity servo performance in both horizontal and vertical directions, accompanied by relatively uniform pressure fluctuations. In both the horizontal and vertical directions, the steady-state error between the measured velocity curve and the desired input remains at a low level. These signify enhanced stability in footplate–ground contact. However, for both the original structure and the group with an inflation pressure of 10/10 bar, the horizontal velocity exhibits pronounced overshoot during the reversal phase. In the comparison of horizontal velocity curves, at the velocity turning points the maximum overshoot relative to the desired input is approximately 7.5%. And in the vertical direction, the maximum fluctuation amplitude increases nearly fivefold compared with the original structure and the low inflation pressure groups. Meanwhile, the pressure fluctuations in the working chamber exhibited minimal amplitude and irregular distribution. This indicates that under the current load conditions, the new structure exhibits instability in ground contact at higher inflation pressures, which can be attributed to the excessive torsional stiffness of the footplate. The overshoot in horizontal velocity is precisely due to the higher torsional stiffness of the footplate, which transforms the originally pure rotational motion of the lower-leg around the spherical hinge into a rolling motion around the contact zone. The integrated horizontal and vertical velocity profiles demonstrate that, although elevated inflation pressure inhibits complete ground contact of the footplate, no overturning is observed during motion. This unequivocally confirms the enhanced stability of the new structure.

**Figure 13:**
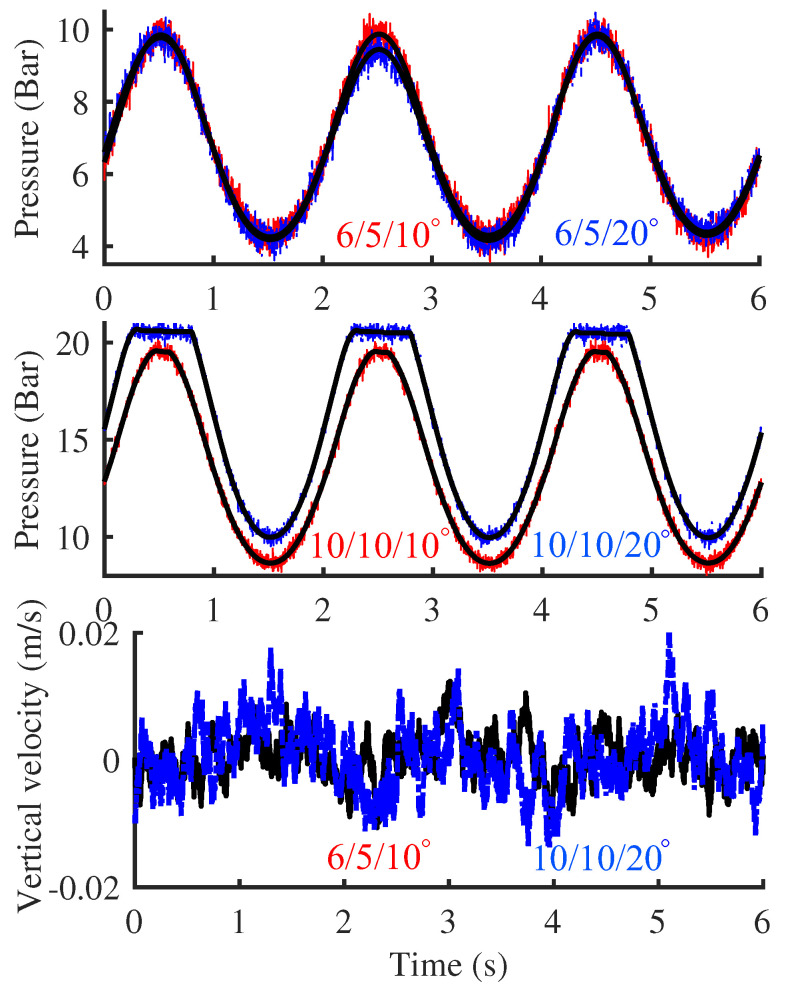
Dynamic response of observation point velocity and chamber pressure under limb lateral tilt.

To further evaluate the applicability of the new structure in quadruped robots across diverse postures, the single-leg module was laterally tilted to $10^{\circ }$ and $20^{\circ }$ by actuating the lateral swing motor, and the preprogrammed motion trajectory mentioned above was subsequently executed, as shown by Figure 13. The curves continue to utilize distinct color-coded numerical combinations to differentiate between inflation pressure and tilt angle. The results show that, under low inflation pressures or small tilt angles, the pressure fluctuations within the working chamber exhibit a distinct and regular periodic pattern. Concurrently, the vertical velocity fluctuations of the single-leg module are notably subdued, which indicates that the contact between the footplate and the ground is also relatively stable. Compared with the previous 10/10 bar experimental group, the footplate had already undergone a rotation relative to its original position upon inclined ground contact, as also evidenced by the range of variation in chamber pressure.

At a tilt angle of 20 degrees, the increased deflection of the footplate’s posture leads to a greater initial compression of the working chamber. As a result, with the inflation pressure held at 10/10 bar, the pressure curve during dynamic motion demonstrates a saturation effect, characterized by a horizontal plateau. This saturation indicates that the footplate has lost contact with the ground. This phenomenon is further corroborated by the velocity curve, which exhibits periodic fluctuations that are perfectly synchronized with the variations in gas pressure.

**Figure 14:**
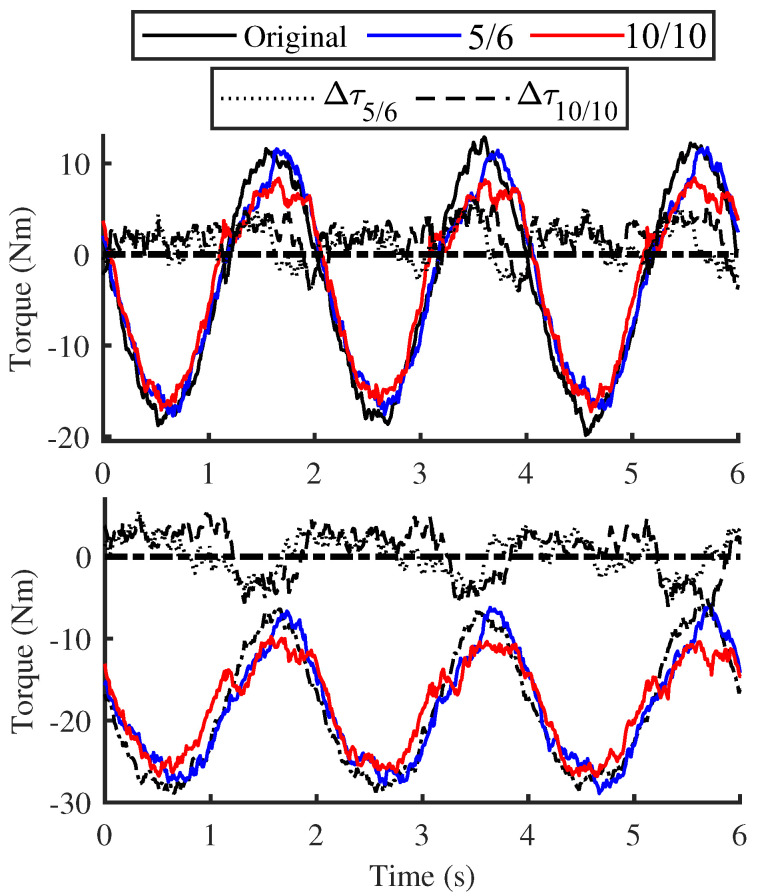
Joint torque comparison with no lateral tilt.

**Figure 15:**
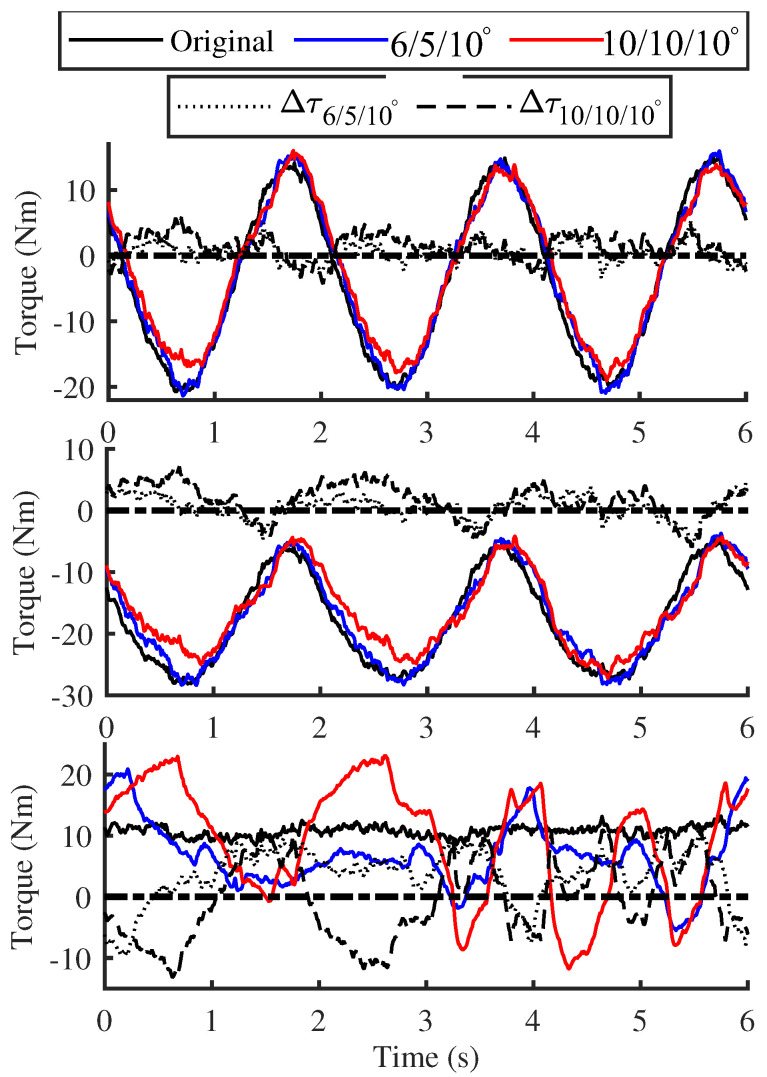
Joint torque comparison with lateral tilt.

The Figure 14 and Figure 15 present the output force of joint motors calculated based on current feedback, in which only two relatively stable data sets under inclined postures were selected for comparison. For ease of comparison, the figure illustrates the differences in the absolute joint torques between the original and new structural groups, where a positive value indicates that the joint torque of the original structural group is slightly larger. The results indicate that, compared to the traditional structure, the driving torques of the leg joints under both postures exhibit a decreasing trend when the new structure is applied, particularly during the motion circumferential phase.

Given the constraints of the overall mass of the single-leg module, initial inflation pressure, motion planning, etc., the magnitude of torque reduction is not substantial. Nevertheless, it is sufficient to demonstrate that the new structure can also contribute to improving the motion energy efficiency of the robot to a certain extent.

## 5. Conclusions and Future Work

This paper proposes a parallel bionic ankle design based on a 3-CUU-S joint configuration to address the limitations legged robot ankle design. By integrating a pneumatic-driven three-piston elastomer with symmetrically arranged, it enables restoration and locking of the footplate pose in the unloaded state, while providing compliant posture adaptation under an external load. Based on the dynamic mathematic model and theoretic analysis, the displacement of piston rods in each adjustment chain and the corresponding variation of the footplate pose are identified, along with their functional relationship to the dynamic response of the elastic element. The performance of the proposed structure is evaluated through experiments conducted on a single-leg testing platform and some conclusions can be drawn as follows:

(1) The footplate–ground interaction stability is governed by the pre-inflation pressure of the elastic element. At relatively high pre-inflation pressures, the footplate remains stable with respect to the main support rod and does not tip over, even when intermittent deflection or separation from the ground occurs.
(2) Under stable foot–ground interaction conditions, the calf undergoes pure rotation relative to the spherical joint on the footplate during forward stepping. Consequently, the single-leg module with the proposed structure exhibits smoother horizontal and vertical motion velocities, with its actual trajectory more closely matching the planned path.
(3) When the restoring torque generated by each posture-adjusting chain at the spherical joint of the footplate aligns with the direction of calf motion, the proposed structure can effectively reduce the driving torque required at the leg joints. Naturally, the extent to which the new structure improves joint driving torque depends on various factors, including stance height, step length, and foot-end trajectory, etc.

Building upon the existing ankle–foot topology, future work will primarily focus on to carry out multi-objective structural optimization design and to investigate ground surface perception mechanisms.

## Figures and Tables

**Table 1:** Structural parameters of flexible foot-ankle.

Symbol	Value	Unit
*H*	258.0	mm
*R*	11.5	mm
$r_{1}$	35.5	mm
$r_{2}\left(r_{3}\right)$	30.8	mm
$\theta_{2}\left(\theta_{3}\right)$	1.7582 (−1.7582)	rad
$l_{1}$	90.0	mm
$l_{2}\left(l_{3}\right)$	63.0	mm

**Table 2:** Elastic element structural and performance parameter specification.

Symbol	Value	Unit	Symbol	Value	Unit
$L_{p0}$	30	mm	$l_{2}\left(l_{3}\right)$	63.0	mm
$L_{r0}$	30	mm	$F_{s}$	100	N
$d_{p}$	8	mm	$F_{c}$	50	N
$d_{r}$	5	mm	$V_{s}$	0.001	m/s
$d_{s}$	12	mm	δ	0.2	-
$l_{1}$	90.0	mm	$\sigma_{v}$	60	-

## Data Availability

The authors confirm that the data supporting the findings of this study are available within the article.

## References

[1] Nguyen Q., Powell M.J., Katz B., Di Carlo J., Kim S. Optimized jumping on the mit cheetah 3 robot. Proceedings of the 2019 International Conference on Robotics and Automation (ICRA).

[2] Villarreal O., Barasuol V., Wensing P.M., Caldwell D.G., Semini C. MPC-based controller with terrain insight for dynamic legged locomotion. Proceedings of the 2020 IEEE International Conference on Robotics and Automation (ICRA).

[3] Yang C., Yuan K., Zhu Q., Yu W., Li Z. (2020). Multi-expert learning of adaptive legged locomotion. Sci. Robot..

[4] Thor M., Kulvicius T., Manoonpong P. (2020). Generic neural locomotion control framework for legged robots. IEEE Trans. Neural Netw. Learn. Syst..

[5] Wang L., Li J. Balance Control of Quadruped Robot under External Disturbance using Quadratic Programming and Impedance Control. Proceedings of the 2023 5th International Conference on Intelligent Control, Measurement and Signal Processing (ICMSP).

[6] Chen G., Hou B., Guo S., Wang J. Dynamic balance and trajectory tracking control of quadruped robots based on virtual model control. Proceedings of the 2020 39th Chinese Control Conference (CCC).

[7] Jeon J., Jeong M., Myung H. Fuzzy Logic and Neural Network-Based Intelligent Control System for Quadruped Robot on Extreme Terrain. Proceedings of the 2023 23rd International Conference on Control, Automation and Systems (ICCAS).

[8] Ma B., Liu Z., Peng C., Li X. Trotting gait control of quadruped robot based on Trajectory Planning. Proceedings of the 2021 4th World Conference on Mechanical Engineering and Intelligent Manufacturing (WCMEIM).

[9] Zhang C., Jin J., Frey J., Rudin N., Mattamala M., Cadena C., Hutter M. Resilient legged local navigation: Learning to traverse with compromised perception end-to-end. Proceedings of the 2024 IEEE International Conference on Robotics and Automation (ICRA).

[10] Chen T., Huangfu Y., Srigrarom S., Khoo B.C. (2024). Path Planning and Motion Control of Robot Dog Through Rough Terrain Based on Vision Navigation. Sensors.

[11] Miki T., Lee J., Hwangbo J., Wellhausen L., Koltun V., Hutter M. (2022). Learning robust perceptive locomotion for quadrupedal robots in the wild. Sci. Robot..

[12] Zhang Z., Chang X., Ma H., An H., Lang L. (2022). Model predictive control of quadruped robot based on reinforcement learning. Appl. Sci..

[13] Spröwitz A., Tuleu A., Vespignani M., Ajallooeian M., Badri E., Ijspeert A.J. (2013). Towards dynamic trot gait locomotion: Design, control, and experiments with Cheetah-cub, a compliant quadruped robot. Int. J. Robot. Res..

[14] Weinmeister K., Eckert P., Witte H., Ijspeert A.J. Cheetah-cub-S: Steering of a quadruped robot using trunk motion. Proceedings of the 2015 IEEE International Symposium on Safety, Security, and Rescue Robotics (SSRR).

[15] Miyashita K., Masuda Y., Gunji M., Fukuhara A., Tadakuma K., Ishikawa M. Emergence of swing-to-stance transition from interlocking mechanism in horse hindlimb. Proceedings of the 2020 IEEE/RSJ International Conference on Intelligent Robots and Systems (IROS).

[16] Shen F., Du R., Nie D., Huang Z., Tian J., Gu J. Design of An Ankle-Foot System with Uneven Terrain Adaptability. Proceedings of the 2022 12th International Conference on CYBER Technology in Automation, Control, and Intelligent Systems (CYBER).

[17] Huang W., Xiao J., Zeng F., Lu P., Lin G., Hu W., Lin X., Wu Y. (2021). A quadruped robot with three-dimensional flexible legs. Sensors.

[18] Jiang Z., Wang Y., Zhang K. (2024). Development of a pneumatically actuated quadruped robot using soft–rigid hybrid rotary joints. Robotics.

[19] Bjelonic F., Lee J., Arm P., Sako D., Tateo D., Peters J., Hutter M. (2023). Learning-based design and control for quadrupedal robots with parallel-elastic actuators. IEEE Robot. Autom. Lett..

[20] Jiang L., Xu Z., Zheng T., Zhang X., Yang J. (2024). Research on Dynamic Modeling Method and Flying Gait Characteristics of Quadruped Robots with Flexible Spines. Biomimetics.

[21] Singh S., Dutta A. (2024). Gait planning and optimization of an 18 DOF quadruped robot with compliant shanks. Proc. Inst. Mech. Eng. Part C J. Mech. Eng. Sci..

[22] Sun Y., Zong C., Pancheri F., Chen T., Lueth T.C. (2023). Design of topology optimized compliant legs for bio-inspired quadruped robots. Sci. Rep..

[23] Zhu Z., Zhu W., Zhang G., Chen T., Li Y., Rong X., Song R., Qin D., Hua Q., Ma S. (2023). Design and control of BRAVER: A bipedal robot actuated via proprioceptive electric motors. Auton. Robot..

